# Supplementary material for: Alleviation of Lipid Disorder and Liver Damage in High-Fat Diet-Induced Obese Mice by Selenium-Enriched Cardamine violifolia with Cadmium Accumulation
Source: Nutrients. 2024 Sep 22;16(18):3208. doi: 10.3390/nu16183208 (PMC11435019; doi:10.3390/nu16183208)
Supplement: Supplementary file 1 [file nutrients-16-03208-s001.zip › nutrients-3208337-supplementary.pdf]

Alleviation of lipid disorder and liver damage in high-fat diet-induced obese mice by selenium-enriched *Cardamine violifolia* with a high cadmium accumulation

Junying Zhu[1#], Qingqing Lv[1#], Fengna Li[1#], Ping Xu[1], Ziyu Han[1], Aolin Yang[1], Zhan Shi[1], Chao Wang[2], Jie Jiang[2], Yunfen Zhu[3], Xiaofei Chen[3], Lvhui Sun[4], Xin Gen Lei[5]*, Ji-Chang Zhou[1,6,7]*

[1]School of Public Health (Shenzhen), Shenzhen Campus of Sun Yat-sen University, Shenzhen, Guangdong 518107, China;

[2]Shenzhen Center for Disease Control and Prevention, Shenzhen, Guangdong 518055, China;

[3]Enshi Autonomous Prefecture Academy of Agricultural Sciences, Enshi, Hubei 445000, China;

[4]Hubei Hongshan Laboratory, College of Animal Science and Technology, Huazhong Agricultural University, Wuhan, Hubei 430070, China;

[5]Department of Animal Science, Cornell University, Ithaca, NY 14853, USA;

[6]Guangdong Province Engineering Laboratory for Nutrition Translation, Shenzhen, Guangdong 518107, China;

[7]Guangdong Provincial Key Laboratory of Food, Nutrition and Health, Guangzhou, Guangdong 510080, China.

# Equal contribution.

*Corresponding authors: JCZ (zhoujch8@mail.sysu.edu.cn) and XGL (xl20@cornell.edu).

Number of tables: 2

Number of figures: 3

Number of pages: 7

Table S1. Primer sequences for qPCR

Gene	Forward, 5’ - 3’	Reverse, 5’ - 3’
Abca1	CCAACATCTGAAAAACAGGTTTGGA	GACACTTCCCGGAAACGCAA
Abcg1	CTACATCTCCTATGTCAGATACGGC	ACTTCTGGAAGTGGCATGTCT
Abcg5	CCTGAACTTCACTTGTGGTGGA	TCTCGATGAACTGGACCCCT
Abcg8	CATCCTCGGAGACACGATGA	ACAGGAAGCCGTAGCTGATG
Actb	ACTGCCGCATCCTCTTCCT	GCCACAGGATTCCATACCCAA
Atgl	ACCATCTGCCTTCCAGACTGT	TAGGGCCTCACTGTAGGTGG
Dgat1	CATGATGGCTCAGGTCCCAC	ATACATGAGCACAGCCACCG
Dgat2	CATCACCACCGTCGTGGG	TCAAAGAGCTTCACCAGGGC
Dio1	CCTGTGGTGGTGGACACAAT	GGGCCAGCTTTACCCTTGTA
Dio3	AAGGGGTGGAAGAGGGACAT	TGCGAACCTCGCAGATTGAT
Gpat1	AAGGACATCGGGGTTTTCAA	GAGAGATCGCTACAGCACCA
Gpat2	TCAAAGATCTGGGGGTACTTCA	TCCAGCTTGTCCTGATTGTCC
Gpx1	CTCTGAGGCACCACGATCC	TTGCCATTCTCCTGGTGTCC
Gpx4	TTGATAAGAACGGCTGCGTG	AGGTCCTTCTCTATCACCTGGG
Hmgcr	AAGAATCTTGTGGGCTCGGC	ACATTCTGTGCTGCATCCTGG
Hsl	CTGTGCACCTTGTGGCTTG	GCCATGCGGCAGATCTTCTA
Ldlr	CCTACCCCTCAAGACAGATGGT	AAGCTTGGCAATGGATCCCG
Lipc	GGAGAGACGCAGCAAAGAATG	AAGACTTTCTCCTGGCTCGG
Lpl	AAGAAGTCTGGCTGACACTGG	AACCCACTTTCAAACACCCAA
Msrb1	GGCCACGAGTTCCTGAATGA	GCAGTCCATGTCCTAGTGCC
Mt1	TCCTGCAAGAAGAGCTGCTG	GTTCGTCACATCAGGCACAG
Mt2	TCCTGCAAGAAAAGCTGCTG	GCACTTGTCGGAAGCCTCTT
Mtf1	CAATGTTCCAGAATTCCGACGA	CTGTCAATCAAGGCAGCTGTC
Selenof	ACAGATCAAGTATGTTCGAGGC	CTCGCTCAGGAACTCTTCCA
Selenoh	AACAGCCGTGTTGAACTCTGGA	TGAGGACTCTTTCCCAACCTCT

Gene	Forward, 5' - 3'	Reverse, 5' - 3'
Selenoi	TCTCCTCTTGGTTGTGGCAG	GCTTCACCACTTGTACGCCA
Selenok	GCTGGTGGATGAGGAAGGTAAA	CTCTTCACCGCTTGATGGCT
Selenom	GTGATGAAGCACCTCCCTGG	GGGATTCGCTCTAGTTCCTGG
Selenoo	GAGACTTTTCAGAGGTGCGG	TTGCTCCTCAGTGCTCCTTG
Selenop	TGGCCGTCTTGTGTATCACC	TCAAGACTCGTGAGATTGCAG
Selenos	CCTTTGCGAGGAGGTGGTTA	AGTTTCAGCCGCCAGATGAT
Selenot	GCACCGAAAACTCTTTTACACT	CAACGAGCCTGCCAAGAAAC
Selenow	ACTGGTGACCGCCATCAAAG	GAGCTTTCAGGACCCTGCCT
Sephs2	GCCACCTGACTTGGTCTCTG	TTCAGCCTCTCCCAGTCCTT
Sqle	TGGGTTGCTTTCAATATTGTCTC	GGCGTAGATTGCAACGGAAA
Txnrd1	CCGGTCTGTGCAGAGATATTCA	GCTTAACCTCAGCAGCCAGA
Txnrd2	GGATCAAGTGTGGGGCTTCA	GCAACCAGTCACAGTAGGCT
Txnrd3	CCACCTGTGGTGAGGTATTCA	TGACAGGGAAACCCAACAGC

Abca1, ATP binding cassette subfamily A member 1; *Abcg1/ 5/ 8*, ATP binding cassette subfamily G member 1/ 5/ 8; *Actb*, actin beta; *Atgl*, adipose triglyceride lipase; *Dgat1/ 2*, diacylglycerol O-acyltransferase 1/ 2; *Dio1/ 3*, iodothyronine deiodinase 1/ 3; *Gpat1/ 2*, glycerol-3-phosphate acyltransferase 1/ 2; *Gpx1/ 4*, glutathione peroxidase 1/ 4; *Hmgcr*, 3-hydroxy-3-methylglutaryl coenzyme A reductase; *Hsl*, hormone-sensitive lipase; *Ldlr,* low density lipoprotein receptor; *Lipc*, lipase C hepatic type; *Lpl*, lipoprotein lipase; *MsrB1*, methionine sulfoxide reductase B1; *Mtf1*, metal regulatory transcription factor 1; *Mt1/ 2*, metallothionein 1/ 2; *Selenof/ h/ i/ k/ m/ o/ p/ s/ t/ w*, selenoprotein F/ H/ I/ K/ M/ O/ P/ S/ T/ W; *Sephs2*, selenophosphate synthetase 2; *Sqle*, squalene epoxidase; *Txnrd1/ 2/ 3*, thioredoxin reductase 1/ 2/ 3.

Table S2. Target protein, type, dilution, and source of primary antibodies used for the Western blot analyses.

Target protein	Source	Isotype	Dilution	Brand (Cat. #)
Actb	Mouse	IgM	1:5,000	Proteintech (60008-1-Ig)
Dio1	Rabbit	IgG	1:2,000	Proteintech (11790-1-AP)
Dio3	Rabbit	IgG	1:2,000	Bioss (Bs-3902R-A488)
Gpx1	Rabbit	IgG	1:1,000	Abcam (ab22604)
Gpx4	Rabbit	IgG	1:5,000	Abcam (ab125066)
Mt	Rabbit	IgG	1:1,000	Abnova (MAB9787)
Mtf1	Rabbit	IgG	1:1,000	Boster (A04733-3)
Selenof	Rabbit	IgG	1:5,000	Abcam (ab124840)
Selenoh	Rabbit	IgG	1:1,500	Abcam (ab151023)
Selenoi	Rabbit	IgG	1:1,000	Abcam (ab157571)
Selenoo	Rabbit	IgG	1:5,000	Abcam (ab172957)
Selenop	Rabbit	IgG	1:1,000	Abcam (ab109514)
Selenos	Rabbit	IgG	1:1,000	Proteintech (15591-1-AP)
Sod1	Rabbit	IgG	1:200	Santa Cruz Biotechnology (sc-11407)
Txnrd1	Rabbit	IgG	1:3,000	Proteintech (11117-1-AP)
Txnrd2	Rabbit	IgG	1:3,000	Proteintech (16360-1-AP)

Actb, actin beta; Dio1/ 3, iodothyronine deiodinase 1/ 3; Gpx1/ 4, glutathione peroxidase 1/ 4; Mt, metallothionein; Mtf1, metal regulatory transcription factor 1; Selenof/ h/ i/ o/ p/ s, Selenoprotein F/ H/ I/ O/ P/ S; Sod1, superoxide dismutase 1; Txnrd1 /2, thioredoxin reductase 1/ 2.

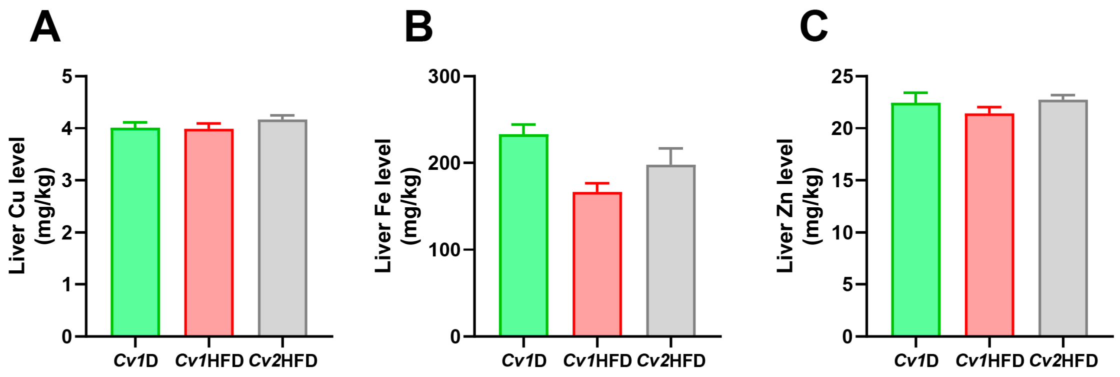

Figure S1. Accumulation of copper (Cu) (A), iron (Fe) (B), and zinc (Zn) (C) in the liver of adult female mice. Data are means ± SEM (*n* = 10 - 12) without differences, $P > 0.05$. *Cv1*D, control diet with 0.15 mg Se and 0.03 mg Cd (in the form of control *Cv*)/kg; *Cv*, *Cardamine violifolia*; *Cv1*HFD, high-fat diet with 0.15 mg Se and 0.03 mg Cd (in the form of control *Cv*)/kg; *Cv2*HFD, high-fat diet with 1.5 mg Se and 0.29 mg Cd (in the form of Se-enriched *Cv*)/kg.

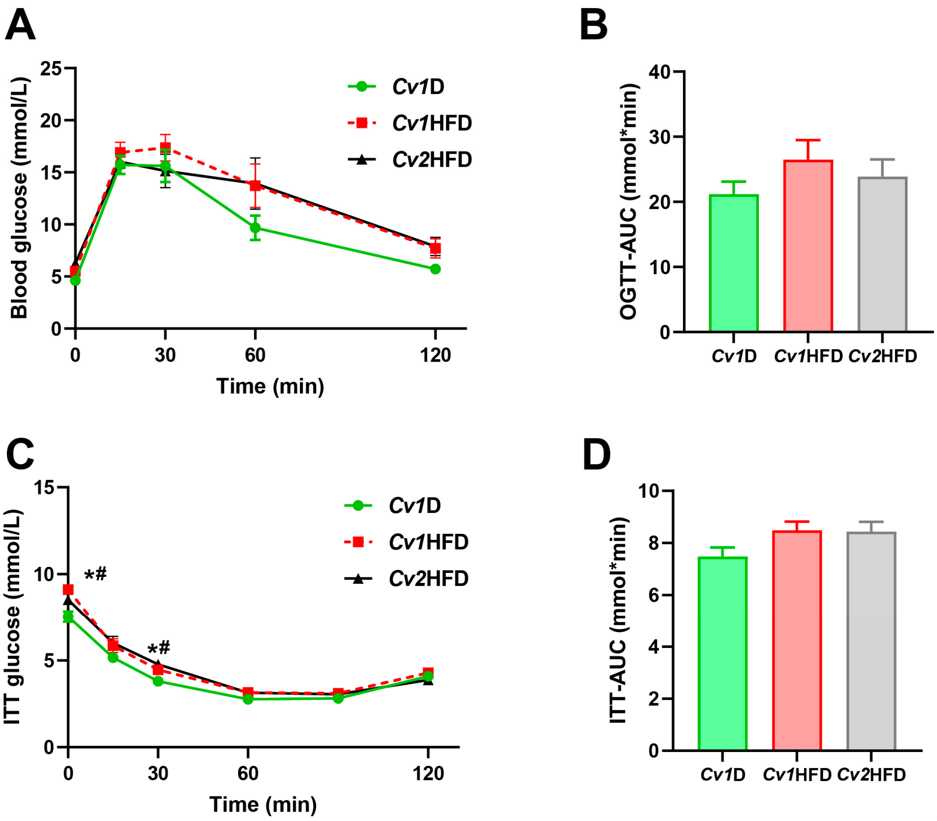

Figure S2. Blood glucose changes in oral glucose tolerance test (OGTT) (A - B) and insulin tolerance test (ITT) (C - D) on adult female mice. Data are means ± SEM (n = 10) and differ with * between the *Cv1*D and *Cv1*HFD groups or # between the *Cv1*D and *Cv2*HFD groups, $P < 0.05$. AUC, area under curve; *Cv1*D, control diet with 0.15 mg Se and 0.03 mg Cd (in the form of control *Cv*)/kg; *Cv*, *Cardamine violifolia*; *Cv1*HFD, high-fat diet with 0.15 mg Se and 0.03 mg Cd (in the form of control *Cv*)/kg; *Cv2*HFD, high-fat diet with 1.5 mg Se and 0.29 mg Cd (in the form of Se-enriched *Cv*)/kg; ITT, insulin tolerance test; OGTT, oral glucose tolerance test.

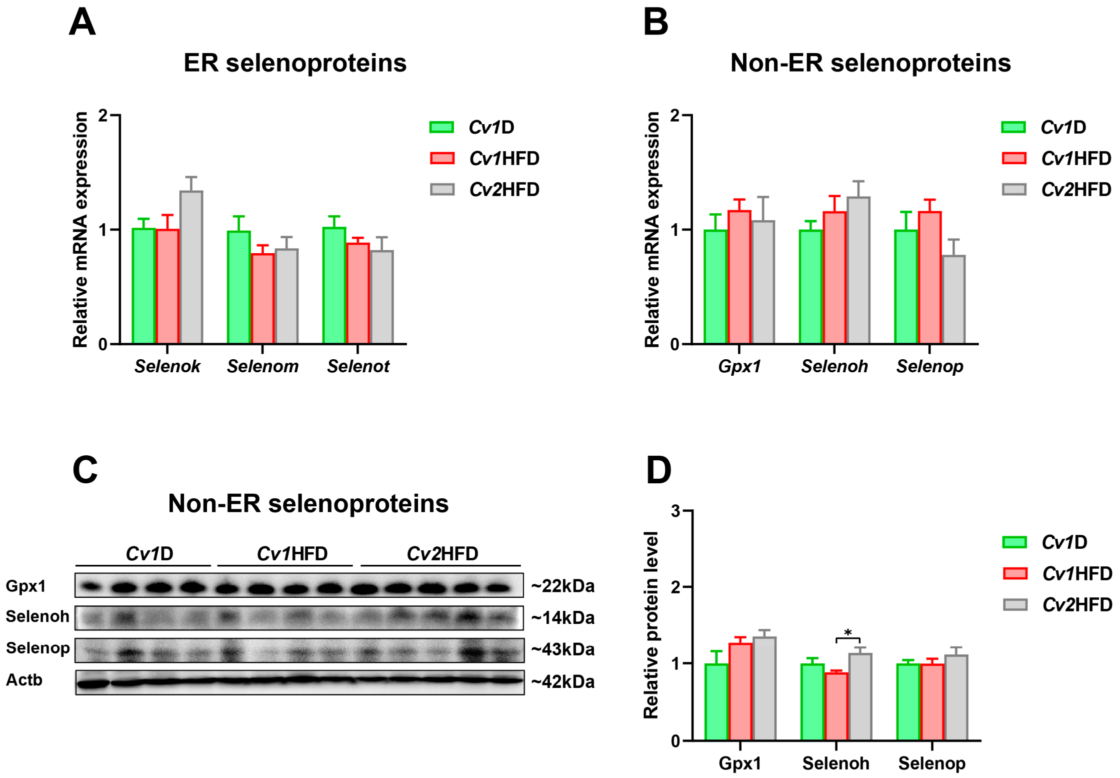

Figure S3. The relative mRNA expression of three endoplasmic reticulum (ER) selenoproteins (A) and three non-ER selenoproteins. (B) in the liver of female mice. (C) A representative immunoblot image for Gpx1, Selenoh and Selenop. (D) The relative protein levels of Gpx1, Selenoh and Selenop. Data are means ± SEM (n = 8 - 10) and differ with *, $P < 0.05$. *Cv1*D, control diet with 0.15 mg Se and 0.03 mg Cd (in the form of control *Cv*)/kg; *Cv*, *Cardamine violifolia*; *Gpx1*, glutathione peroxidase 1; *Cv1*HFD, high-fat diet with 0.15 mg Se and 0.03 mg Cd (in the form of control *Cv*)/kg; *Cv2*HFD, high-fat diet with 1.5 mg Se and 0.29 mg Cd (in the form of Se-enriched *Cv*)/kg; *Selenoh /k /m /p / t*, selenoprotein h /k /m /p /t.
